# The Roles of N6-Methyladenosine Modification in Plant–RNA Virus Interactions

**DOI:** 10.3390/ijms242115608

**Published:** 2023-10-26

**Authors:** Min He, Zhiqiang Li, Xin Xie

**Affiliations:** 1 Laboratory of Agricultural Microbiology, College of Agriculture, Guizhou University, Guiyang 550025, China; ming19128@163.com; 2 State Key Laboratory for Biology of Plant Diseases and Insect Pests, Institute of Plant Protection, Chinese Academy of Agricultural Sciences, Beijing 100193, China; zhiqiangdo_771@163.com

**Keywords:** m^6^A, RNA virus, infection, plant

## Abstract

N^6^-methyladenosine (m^6^A) is a dynamic post-transcriptional RNA modification. Recently, its role in viruses has led to the study of viral epitranscriptomics. m^6^A has been observed in viral genomes and alters the transcriptomes of both the host cell and virus during infection. The effects of m^6^A modifications on host plant mRNA can either increase the likelihood of viral infection or enhance the resistance of the host to the virus. However, to date, the regulatory mechanisms of m^6^A in viral infection and host immune responses have not been fully elucidated. With the development of sequencing-based biotechnologies, the study of m^6^A in plant viruses has received increasing attention. In this mini review, we summarize the positive and negative consequences of m^6^A modification in different RNA viral infections. Given its increasingly important roles in multiple viruses, m^6^A represents a new potential target for antiviral defense.

## 1. Introduction

There are currently more than 150 known types of RNA modification. The most widespread type of RNA modification is methylation including N^6^-methyladenosine (m^6^A), 5-methylcytosine (m^5^C), N^7^-methylguanosine (m^7^A), and N^1^-methyladenosine (m^1^A) [1]. m^6^A is a type of methylation that occurs on the N-atom at the sixth position of adenine (A) bases [2]. The underlying methylation reaction, which affects almost every stage of mRNA metabolism [3], represents the most prevalent internal modification of eukaryotic mRNA, accounting for over 80% of all RNA base methylations in various species [4]. RNA m^6^A methylation is a dynamically reversible modification within the cell that is regulated by methyltransferases, demethylases, and m^6^A binding proteins [5,6]. The effects of m^6^A modification include the functional modulation of mRNA splicing, export, localization, translation, and stability by regulating RNA structure and interactions between RNA and RNA-binding proteins [7,8,9,10].

Since the 1970s, m^6^A modification has been known to tag not only cellular RNA but also the RNA of multiple viruses [11]. In the following decades, m^6^A modifications were identified in viral RNA in mammals, including simian virus 40 (SV40) [12], Rous sarcoma virus (RSV) [13], and Influenza A virus (IAV) [14]. However, the functional relevance of m^6^A modification has remained elusive, mainly due to the lack of efficient methods of detection and subsequent analysis. Recent studies have demonstrated the crucial roles of m^6^A in virus–host interactions [15,16,17]. The dynamics of m^6^A modifications, including the precise locations, frequency of methylation, and percentage of methylated genes, may differ in plants subjected to biotic and abiotic stress, particularly plants infected with viruses. Although m^6^A modification plays a crucial role in controlling the viral life cycle and reproduction in animal systems, researchers still have a limited understanding of its significance in plant viruses [17,18,19].

Plants are infected by many viruses over the course of their growth, including by double- and single-stranded DNA and RNA viruses. However, the genome of most plant viruses is RNA based. These viruses cause severe diseases in numerous crops worldwide, resulting in substantial losses to agricultural production [20]. Plant RNA viruses are classified based on their genome composition, like the single-stranded RNA positive-strand viruses from the families Potyviridae (potato virus Y [PVY]), Bromoviridae (alfalfa mosaic virus [AMV], and cucumber mosaic virus [CMV]), single-stranded RNA negative-strand viruses from the Ophioviridae family, and double-stranded RNA viruses from the Partitiviridae family [21]. The majority of plant viruses (~80%) contain single-stranded RNA genomes ranging in size from 2.5 to 10 kb, with the majority being in the range from 4 to 6 kb. During the viral infection multiple symptoms occur, the vast majority of which are host-specific [22]. The symptoms of viral infection occur as a result of complex interactions between the virus and its host plant. With advances in our understanding of RNA viruses and the development of sequencing technology, the roles of m^6^A modification in modulating viral infection are now being uncovered [23]. The frequency of m^6^A modification in tobacco (*Nicotiana tabacum*) exhibited a significant reduction subsequent to infection with tobacco mosaic virus (TMV) [18]. This discovery implies that the m^6^A modification could serve as a regulatory mechanism utilized by plants to effectively counteract viral infections. Significantly, the genomes of various single-stranded RNA plant viruses have been discovered to contain a conserved alkylation B domain sequence, which is a component of m^6^A demethylases [24,25]. For example, members of the family Flexiviridae, including Grapevine virus A (GVA), Blueberry scorch virus (BlScV), and Blackberry virus Y (BVY), as well as an unnamed new genus in the family Potyviridae, contain ALKB-like domains. Sequence analysis revealed that the ALKB domain may be involved in the repair of methylated RNA damage and plays an important role in clearing the viral genome of harmful RNA and maintaining the stability of viral RNA [26]. These findings confirm that some plant viruses have developed responses to this mechanism in the host, and that m^6^A modification may be a technique used by plants to fine-tune their responses to viral infection.

The structural diversity and functional characteristics of RNA genomes in most plant viruses pose challenges for their comprehensive characterization using conventional molecular biology methodologies [27]. Recent developments in high-throughput sequencing methods for m^6^A have facilitated functional research on this RNA modification. Two major methods are currently used to identify m^6^A modifications.

The first method is antibody-dependent m^6^A sequencing. (i) m^6^A antibody affinity enrichment combined with high-throughput sequencing (methylated RNA immunoprecipitation followed by sequencing [MeRIP-seq] or m^6^A-seq) was the first high-throughput sequencing technique developed using m^6^A antibodies. MeRIP-seq/m^6^A-seq is simple to perform, and all reagents have been commercialized; thus, it has always been the first choice for m^6^A sequencing [28,29]. (ii) In m^6^A individual-nucleotide-resolution cross-linking and immunoprecipitation (m^6^A-CLIP/miCLIP), the two methods cannot accurately locate m^6^A in multiple adjacent adenines, and the analysis of clustered m^6^A distribution is difficult [30,31]. (iii) An upgraded MeRIP-seq/m^6^A-seq method (m^6^A-seq2): performs, all m^6^A-IPs in a single tube, which can lower technical variability, starting material requirements, and library preparation costs [32].

The second method is antibody-independent m^6^A sequencing, including (i) m^6^A selective chemical labeling using the m^6^A demethylase Fat mass and obesity-associated protein (FTO) (m^6^A-SEAL-seq), in which the amount of initial RNA required for m^6^A-SEAL-seq is low, and there is almost no sequence selectivity. The disadvantages of this method are that it involves many operations and takes a long time [33]. (ii) In selective allyl chemical labeling and sequencing (m^6^A-SAC-seq), the reaction is based on an enzyme and may therefore have a sequence preference [34]. (iii) Glyoxal and nitrite-mediated deamination of unmethylated adenosine and sequencing (GLORI-seq) enables efficient and unbiased detection of single base m^6^A sites and absolute quantification of m^6^A modification level [35].

The rapid development of these sequencing technologies has advanced the study of plant–virus interactions. However, the molecular functions of this modification, the dynamics of m^6^A in plant–virus interactions, and the relationship between the expression levels of important disease resistance pathway–related genes in the host and the m^6^A modification regions on gene bodies in the host are all still unknown. Furthermore, plants have evolved sophisticated systems for detecting and battling viruses after a plant has been infected by a virus, including protein degradation, RNA silencing, immune receptor signaling, and hormone-mediated defense pathways. The two primary examples of plant defense systems are pathogen-associated molecular patterns (PAMP)-triggered immunity (PTI) and effector-triggered immunity (ETI). It is still unknown whether the m^6^A level of the viral genome is affected as well as whether some of the immune mechanisms ([PTI] and [ETI]) of the plant itself change. In this review, we focus on recent findings on the functions of N^6^-methyladenosine modification in plant–RNA virus interactions. We hope that this review serves as a reference for future in-depth studies of the roles and mechanisms of m^6^A in regulating viral infection.

## 2. Molecular Mechanism of m^6^A Modification

The deposition of m^6^A is achieved by a multicomponent methyltransferase complex [36]. The deposition of the m^6^A modification is regulated by three types of protein, commonly referred to as “writer” (m^6^A methyltransferase), “eraser” (m^6^A demethylase), and “reader” (m^6^A binding) proteins. Writers and erasers perform the reversible deposition and removal of this modification, respectively [37]. The first reports of m^6^A in plant mRNAs date back to 1979, when it was first discovered in wheat (*Triticum aestivum*) and maize (*Zea mays*) [38,39]. Shortly after the discovery of m^6^A in plant mRNAs, scientists identified the RR**A**CH sequence motif (where R = G/A, H = A/C/U, with the bold letter representing the m^6^A-modified adenosine) [17,40]. However, despite this initial interest, research in plant m^6^A decreased until it was rediscovered in Arabidopsis (*Arabidopsis thaliana*) in 2008. In this study, the functional importance of the mRNA adenosine methylase A gene (MTA, an ortholog of human Methyltransferase 3 [METTL3]) was demonstrated through loss-of-function experiments, where disruption of the gene resulted in the death of the plant embryo [41]. Since then, Several additional m^6^A methyltransferases have been identified in plants, including MTB (homolog of human METTL14) [40], VIRILIZER (VIR, homolog of human KIAA1429) [42], FKBP12 Interacting Protein 37 (FIP37, homolog of human WT1-associated protein [WTAP]) [43], HAKAI [41,44], FIONA1 (FIO1, ortholog of human METTL16) [45], FLOWERING LOCUS PA (FPA, homolog of human RNA Binding Motif Protein 15 [RBM15, RBM15B]) [46], and HAKAI Interacting Zinc finger protein 1 and 2 (HIZ1 and HIZ2, homologs of human ZC3H13) [47]. Other such proteins remain to be discovered. The continuous discovery of new m^6^A-related proteins has driven ongoing research in this field.

In contrast to writers and readers, our knowledge about eraser proteins is limited. The alkylation B homolog (ALKBH) protein, a member of the α-ketoglutarate (αKG) and Fe (II) dioxygenase superfamily, removes alkyl and methyl groups from DNA and has been proposed to function as an RNA demethylase [29,48,49]. The functions of only a few eraser proteins in plants have been determined, and 13 Arabidopsis ALKBH family members have been identified by bioinformatics analysis [50,51]. The demethylase activities of ALKBH9B and ALKBH10B have been shown in Arabidopsis, demonstrating the functions of ALKBH9B, ALKBH10B, and ALKBH6. ALKBH10B is an mRNA m^6^A eraser that influences flowering, and ALKBH6 functions in seed germination, seedling growth, and the survival of Arabidopsis under abiotic stress [52,53].

**Figure 1:**
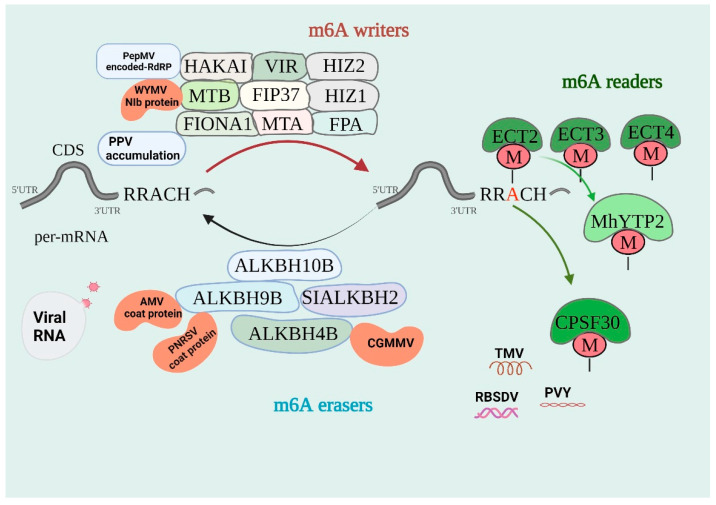
m^6^A methylation pathways and related m^6^A methylated genes in plant-RNA virus interactions. The m^6^A modification is regulated by the “writers”, “erasers”, and “readers”. Writers are MTA, MTB, VIR, HAKAI, FIP37, FIONA1, FPA, and HIZ1-2, which have been reported to induce m^6^A RNA methylation. Among them, HAKAI interacts with RdRp encodes for PepMV, MTB binds to the NIb of WYMV promoting viral infection, and overexpression FIONA1 causes a decrease in PPV. Erasers are m^6^A demethylases, including ALKBH10B, SIALKBH2, ALKBH9B, and ALKBH4B. AtALKBH9B interacts with AMV and PNRSV coat protein; ClALKBH4B could induce CGMMV. Readers are proteins that bind to m^6^A-modified mRNAs and play corresponding roles. Proteins that have been identified as readers to date include ECT2, ECT3, ECT4, CPSF30, and MhYTP2. TMV, PVY, and RBSDV reduce or affect the level of m^6^A methylation. WYMV, wheat yellow mosaic virus; AMV, alfalfa mosaic virus; PNRSV, Prunus necrotic ringspot virus; CGMMV, cucumber green mottle mosaic virus; PVY, Potato virus Y; RBSDV, rice black-stripe dwarf virus; TMV, tobacco mosaic virus; PepMV, pepino mosaic virus; PPV, plum pox virus.

Finally, several proteins that recognize the m^6^A modification have been identified, including reader proteins from the YTH family, named after the YT521-B homology domain they contain [28,54,55]. Plants have thirteen YTH identified family proteins, 11 of which have been designated as Evolutionarily Conserved C-Terminal Region 1–11 (ECT 1–11) and, as a predominant isoform of the polyadenylation factor CLEAVAGE AND POLYADENYLATION SPECIFICITY FACTOR30 (CPSF30), CPSF30-L consists of CPSF30-S and an m^6^A-binding YTH domain; it was identified as a novel m^6^A reader in Arabidopsis. CPSF30-L is the homolog of YTHDC1; it is located in the nucleus and is involved in alternative polyadenylation (APA) regulation [56,57,58]. These studies revealed an additional function for m^6^A in RNA metabolism. An overview of the linked machinery and molecular activities of m^6^A is shown in Figure 1 [59,60].

## 3. Regulation of m^6^A Methylation in Plant RNA Viruses

A growing number of studies have shown that m^6^A and its related proteins play a key role in the viral infection process, and their regulatory role is summarized in Table 1 according to the different virus species. The RNAs of the plant viruses AMV and CMV contain m^6^A methylation. Analysis of the effect of the demethylation activity of Arabidopsis ALKBH9B(AtALKBH9B) on the infectivity of AMV showed that this protein affects the infectivity of AMV, but not CMV. The suppression of AtALKBH9B function increased the relative abundance of m^6^A over the AMV genome, impairing the systemic invasion of the plant while not having any effect on CMV infection [19]. The differences in the effects of AtALKBH9B on these two viruses might be related to the ability of AtALKBH9B to interact with viral coat proteins (CPs). Characterization of viral proteins is crucial for a better understanding of these virus–plant interactions as well as the characterization of host components involved in the infectious process. As already described for various plant viruses, coding regions of viral genomes, regulatory elements, non-coding sequences, or silent mutations may be involved in the induction of viral symptoms [61,62,63]. Indeed, a study to dissect the functional activity of AtALKBH9B in AMV infection indicated that amino acid residues between positions 427 and 467 were critical for the in vitro binding of AtALKBH9B to AMV RNA. The AtALKBH9B amino acid sequence contains intrinsically disordered regions located at its N-terminal region delimiting the internal AlkB-like domain and at the C-terminal region. An RNA-binding domain containing an RGxxxRGG (where R = G/A) motif that overlaps with the C-terminal intrinsically disordered region was identified in AtALKBH9B [64]. Bimolecular fluorescence complementation analysis revealed that residues located between positions 387 and 427 in AtALKBH9B interacted with AMV CP and were likely critical for modulating viral infection. Deleting either the 20 N-terminal residues or the 40 C-terminal residues in this protein impeded the accumulation of short interfering RNA (siRNA) bodies in the plant. This mechanism may represent a component of a previously unknown antiviral system because genetic depletion of a plant m^6^A demethylase negatively affected AMV accumulation and movement. Thus, AtALKBH9B affects the ability of AMV to infect the plant [65]. These findings suggest that m^6^A demethylase activity (AtALKBH9B) plays a role in AMV infection in plants. Further research is needed to systematically explain the molecular mechanism of m^6^A regulation of host and viral RNA during AMV infection.

AMV and CMV belong to the Bromoviridae family, but AtALKBH9B does not interact with the CP of CMV [66]. However, AtALKBH9B interacts with the CP of prunus necrotic ringspot virus (PNRSV), which is functionally and phylogenetically closely related to AMV [67,68]. However, since Arabidopsis is not a host of PNRSV, the infectivity of the virus cannot be tested in plants lacking ALKBH9B function, although AtALKBH9B may regulate the life cycles of other Bromoviridae family viruses. Since m^6^A plays different roles in different types of viral infection in plants, it will be important to study the roles of m^6^A in different viruses and hosts in the future.

**Table 1:** Roles of m^6^A in different viral infections.

Virus Name	Virus Classification	Mechanisms	Reference
AMV	Bromoviridae I family	AtALKBH9B correlates with the ability to interact with AMV coat proteins.	[19,65,66]
PNRSV	Bromoviridae I family	AtALKBH9B was found to interact with the CP of PNRSV (a functionally and phylogenetically closely related AMV).	[67]
PVY	Potyviridae family	PVY in *Nicotiana benthamiana* reduces the level of m^6^A methylation.	[26]
PPV	Potyviridae family	Overexpression of *NbMETTL1* and *NbMETTL2* caused a decrease in PPV accumulation.	[26]
ENMV	Potyviridae family	The P1 of ENMV contains AlkB domains.	[69]
WYMV	Potyviridae family	TaMTB binds to the NIb of WYMV and thus promotes viral infection.	[70,71]
BlVY	Potyviridae family	BIVY has an AlkB domain of RNA demethylase activity.	[24,69]
PepMV	Flexiviridae family	RdRp encoded by PepMV could interact with SlHAKAI and promote its protein degradation.	[72]
TMV	Virgaviridae family	The global m^6^A level was reduced under TMV infection, probably associated with decreased m^6^A methyltransferase and increased demethylase expression.	[18]
CGMMV	Virgaviridae family	ClALKBH4B has a significant induction effect in the early response of resistant watermelon to CGMMV.	[73]
RBSDV	Reoviridae family	The m^6^A methylation is mainly associated with genes that are not actively expressed in virus-infected rice plants.	[74,75]
RSV	Phenuiviridae family		[75]

Plum pox virus (PPV), PVY, and endive necrotic mosaic virus (ENMV) belong to the *Potyvirus* genus. Infection with PPV or PVY reduced the level of m^6^A methylation in *Nicotiana benthamiana*. *NbALKB1* and *NbALKB2* were amplified by Yue et al., who extracted them from cDNA samples obtained from *N. benthamiana* plants [26]. The authors reasoned that overexpressing these newly identified *N. benthamiana ALKBH9B* homologs would affect the systemic movement of PPV. However, no significant variations in PPV accumulation were observed in local (6 days post infection [dpi]) or upper non-inoculated (14 dpi) leaf samples collected from treated and control plants, as determined by immunoblotting with PPV CP-specific antiserum. However, this assay may lack the necessary sensitivity, or endogenous levels of these AlkB homologs may already have been sufficient to reach a PPV fitness maximum. Notably, the authors determined that the P1 region of ENMV contains AlkB domains and identified an additional virus from a putative new species within *Potyvirus* containing these domains [69]. A polyprotein leader of blackberry virus Y (BlVY), an unusual *Potyvirid* from the *Brambyvirus* genus, was found to contain a viral AlkB domain exhibiting RNA demethylase activity [24,76]. Phylogenic analysis revealed that ENMV domains share a common origin, while BlVY AlkB belongs to a divergent branch. These results show that two *Potyviruses* and a *Brambyvirus* possess the AlkB genes, multiple independent gene acquisition events have contributed to the evolution of *Potyvirid* AlkB, and RNA methylation likely plays a significant role in driving the evolution of *Potyvirus*. Further research is needed to fully understand the relationship between RNA methylation and *Potyviruses*. Overexpression of the ALKBH9B homolog in PPV-infected plants did not affect the systemic movement of the virus [26]. Overexpression of NbMETTL1 and NbMETTL2 (related to human METTL16 and Arabidopsis *FIONA1*) caused a decrease in PPV accumulation, indicating that METTL homologs participate in plant antiviral responses [77]. Collectively, these studies demonstrate that demethylase of m^6^A is a common modification in different plants and suggest that m^6^A modification plays essential roles in different plant–RNA virus interactions.

A recent study demonstrated that overexpressing the tomato (*Solanum lycopersicum*) m^6^A writer gene *SlHAKAI* negatively regulated pepino mosaic virus (PepMV) infection and inhibited viral RNA and protein accumulation by affecting viral m^6^A levels in tomato plants. On the contrary, there was a direct interaction observed between SlHAKAI and the RNA-dependent RNA polymerase (RdRp) encoded by PepMV, resulting in a decrease in the accumulation of SlHAKAI. Additionally, it has been found that PepMV RdRp exploits the autophagy pathway by directly interacting with SlBeclin1 to facilitate the autophagic degradation of SlHAKAI [72]. These findings indicate that a viral protein has the ability to exploit an autophagy factor for compromising the m^6^A-mediated antiviral response, which represents a novel strategy employed during the ongoing competition between plants and viruses.

## 4. RNA Viruses Affect m^6^A Methylation

DNA methylation has little effect on RNA viruses due to the absence of DNA during their replication cycle. However, RNA-based m^6^A has a demonstrated ability to control cytoplasmic-replicating viruses, pointing to a new layer of the defense mechanism that promotes viral infection [78]. After the virus enters the host cell, it sheds its coat to release its genomic RNA, which is interpreted by transfer RNAs (tRNAs) and the host ribosome using viral RNA as the template. The codon sequences stored in the viral RNA are converted into amino acid sequences, and the products related to viral genome replication are translated. The proteases of the host plant change in response to viral infection, and several methyltransferases may interact with viral proteins and promote viral infection. During wheat yellow mosaic virus (WYMV) infection, resistant and susceptible wheat varieties exhibit a significant variation in their m^6^A modification patterns. Transcriptome-wide m^6^A profiling of WYMV-infected resistant and susceptible wheat varieties revealed that differential m^6^A modifications may disrupt host–pathogen interaction pathways by regulating the expression of related genes [74,75]. The genes investigated in these studies are closely associated with plant defense mechanisms and resilience against pathogens. As a result, they have been considered as potential genes for investigating the strategies employed by wheat to resist viral infections and for understanding the mechanisms through which viruses effectively invade wheat plants. Translocation of wheat m^6^A methyltransferase B (TaMTB) into cytoplasmic aggregates is facilitated by its interaction with the NIb protein of WYMV. This interaction leads to an increase in the m^6^A levels of WYMV genomic RNA1 and stabilization of viral RNA, thereby facilitating viral infection. TaMTB may function as an m^6^A methyltransferase that relocates to cytoplasmic punctate structures upon binding with WYMV NIb protein, suggesting that m^6^A methyltransferases play a role in viral life cycle and regulation of viral involvement in host antiviral innate immunity [79]. Further experimental investigations are required to validate the regulatory impact of m^6^A RNA modifications on the expression of these candidate genes during plant defense against viral infection.

Viral infection also affects the m^6^A levels of endogenous host RNA. One study reported that the m^6^A level decreased in tobacco following TMV infection [18]. In contrast, a study of high-quality m^6^A methylomes of rice (*Oryza sativa*) plants infected with rice stripe virus (RSV) or rice black-stripe dwarf virus (RBSDV) revealed increased levels of m^6^A in rice RNA following RSV or RBSDV infection; this m^6^A methylation was mainly associated with genes that are not actively expressed in virus-infected rice plants [74]. These findings suggest that the regulation of plant m^6^A by viral invasion is complex. The overall plant m^6^A level is altered by RNA viruses of the genera *Tobamovirus*, *Bymovirus*, *Tenuivirus*, and *Fijivirus* [79]. m^6^A modifications in various models were observed on the same gene, possibly reflecting different disease resistance models after two viral infections. For instance, in RBSDV- and RSV-infected samples, the writer genes *OsMTA3* and *OsMTA4* underwent m^6^A methylation. The eraser genes *OsALKBH10B* and *OsALKBH9B* experienced m^6^A modification only in RSV-infected samples, while no eraser genes showed m^6^A methylation in RBSDV-infected samples. Regarding reader genes, RBSDV-infection led to m^6^A methylation of *OsYTH01*, *OsYTH10*, *OsYTH11*, and *OsYTH12*, whereas RSV-infection resulted in m^6^A methylation of *OsYTH05* and *OsYTH08*. Several antiviral pathway-related genes, such as genes involved in RNA silencing, resistance, and fundamental antiviral phytohormone metabolism, were also m^6^A-methylated. The level of m^6^A methylation is tightly associated with the relative expression level of a gene. These observations highlight the importance of m^6^A modification in plant–virus interactions, especially in regulating the expression of genes associated with key pathways.

The tobamovirus cucumber green mottle mosaic virus (CGMMV) has been considered to be the major global plant virus in cucurbit plants. The induction of fruit decay is among the most severe symptoms and is responsible for significant production losses [73]. To discover the molecular mechanism involved in the induction fruit decay He et al. analyzed the m^6^A methylation spectrum in the response of watermelon (*Citrullus lanatus*) to CGMMV infection, using Gene Ontology analysis combined with a transcriptome deep sequencing (RNA-seq) approach, and analysis of the response patterns and putative functions of differentially expressed and m^6^A-modified genes in the transcriptome of CGMMV-infected watermelon leaves. Research shows that the global m^6^A level in resistant watermelon clearly decreased after CGMMV infection. Both the m^6^A methylation and transcript levels of 59 modified genes significantly changed in response to CGMMV infection; some of these genes are involved in plant immunity. The authors proposed a preliminary hypothesis to explain the mechanism of the resistance of watermelon to viral infection via m^6^A modification: the m^6^A demethylase gene *ClALKBH4B* is significantly induced as an early response to CGMMV in resistant watermelon, and the decreased expression of *ClALKBH4B* results in the methylation of numerous target genes, leading to their downregulation. The m^6^A methylation of transcripts is generally negatively correlated with transcript levels. Therefore, the expression of various downstream defense response factors involved in virus-induced gene silencing, transcription factor genes, and genes involved in plant carbohydrate allocation and signaling is induced (as revealed by RNA-seq) and a series of plant immune responses are activated during the early stage of CGMMV infection. Whether changes in the m^6^A modification of these genes affects viral replication deserves further study.

## 5. Conclusions and Perspectives

m^6^A modification is dynamic, reversible, and widely involved in the modification of host and viral RNA; it modulates complex regulatory mechanisms and diverse activities. m^6^A plays different roles in different viruses, different host cells, and even at different times. m^6^A can directly modify viral RNA, thereby affecting viral gene expression or host immune system recognition. This modification can also indirectly regulate viral infection by regulating expression of host genes, such as genes in the host innate immune pathway, cell metabolic pathways, and other related genes. Although many studies have shown that m^6^A modification has an important regulatory role in the viral life cycle, its regulatory mechanism is not yet clear. Therefore, m^6^A modification in the context of plant–virus interactions remains largely unexplored. There is a need for a better understanding of how m^6^A modification affects the interplay between plant hosts and viral pathogens.

To date, m^6^A methyltransferase and m^6^A demethylase have been shown to function in RNA virus–plant host interactions to affect the host or virus, but there has been no breakthrough in identifying m^6^A binding proteins. In fact, the role of methylation depends on the m^6^A reader protein [55]. m^6^A modification is also involved in the interaction between plants and pathogenic fungi. Guo et al. [80] discovered the role of an m^6^A binding protein (MhYTP2 from Chinese crab apple [*Malus hupehensis*]) in plant–microbe interactions. By accelerating the degradation of the bound mRNAs of *MdMLO19* and *MdMLO19-X1* and increasing the translation efficiency of antioxidant genes, *MhYTP2*, a homolog of *EVOLUTIONARILY CONSERVED C-TERMINAL REGION 2* (*ECT2*), enhanced apple resistance to powdery mildew. *Magnaporthe oryzae* RNA’s m^6^A alteration was the subject of another investigation [81]. The functional significance of the m^6^A alteration for *M. oryzae* infection was highlighted by the fact that MTA1, which is involved in m^6^A modification, is deficient. This results in decreased appressorial penetration, invasive development of *M. oryzae*, and highly disrupted autophagy in the mutant. Thus, m^6^A binding proteins may also influence pathogenic bacteria. In addition, the roles of m^6^A binding proteins in different viruses should be studied.

To fulfill the constantly increasing need for food and feed, innovative agricultural practices are required [82]. In two crops, overexpressing m^6^A demethylase genes increased yield by almost 50%, according to a recent ground-breaking study [83]. New approaches to virus control and epigenetic reprogramming of agricultural attributes might be sparked by improvements in the customized manipulation of RNA methylation by plant genome engineering or viral vector delivery. Indeed, whereas recent studies have demonstrated that changes in m^6^A modification in viral or host RNA can regulate viral infection, the specific regulatory mechanism has not yet been deeply studied. Future research may need to comprehensively consider various factors (e.g., cell type, viral strain and infection time) and use optimal sequencing technologies to systematically analyze the roles and specific mechanisms of virus or host m^6^A in the viral replication cycle to provide a new theoretical basis for antiviral research.
